# 7-Chloro-3-(4-methyl­benzene­sulfon­yl)pyrrolo[1,2-*c*]pyrimidine

**DOI:** 10.1107/S241431462000382X

**Published:** 2020-03-27

**Authors:** Easha Narayan, Liangfeng Fu, Gordon W. Gribble, Manpreet Kaur, Jerry P. Jasinski

**Affiliations:** a Department of Chemistry, Dartmouth College, Hanover, NH 03755 , USA; b Department of Chemistry, Keene State College, 229 Main Street, Keene, NH 03435 , USA; University of Aberdeen, Scotland

**Keywords:** crystal structure, weak inter­molecular inter­actions, pyrrolo­[1,2-*c*]pyrimidine and pyrimidine rings, π–π stacking

## Abstract

In the title compound, C_14_H_11_ClN_2_O_2_S, the dihedral angle between the pyrrolo­[1,2-*c*]pyrimidine ring system (r.m.s. deviation = 0.008 Å) and the benzene ring is 80.2 (9)°. In the crystal, inversion dimers linked by pairs of C—H⋯O inter­actions generate *R* ^2^_2_(16) loops. Several aromatic π–π stacking inter­actions between the pyrrolo­[1,2-*c*]pyrimidine rings, as well as separately between the pyrrolo and pyrimidine groups [shortest centroid–centroid separation = 3.5758 (14) Å], help to consolidate the packing.

## Abstract 2

The dihedral angle between the pyrrolo­[1,2-*c*]pyrimidine ring system and the benzene ring is 80.2 (9)°. In the crystal, inversion dimers linked by pairs of C—H⋯O inter­actions generate 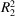 (16) loops. Several aromatic π–π stacking inter­actions between the pyrrolo­[1,2-*c*]pyrimidine rings, as well as separately between the pyrrolo and pyrimidine groups [shortest centroid–centroid separation = 3.5758 (14) Å], help to consolidate the packing.

## Graphical abstract

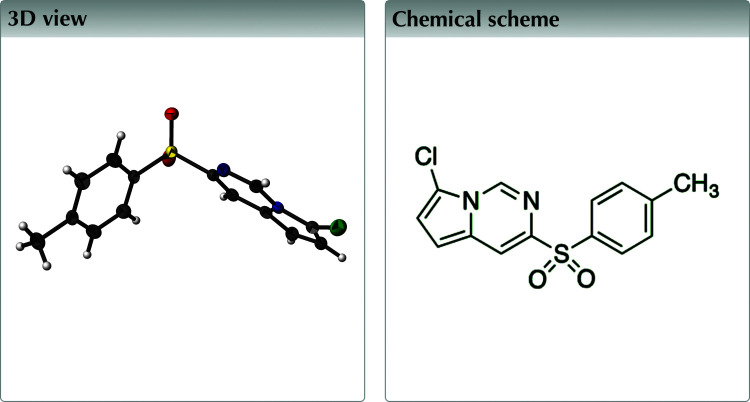

## Structure description

**Figure 1:**
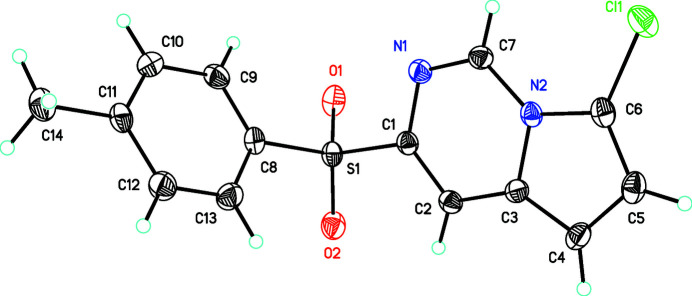
The mol­ecular structure of the title compound, showing 50% probability ellipsoids.

Pyrrolo­[1,2-*c*]pyrimidines are a class of fused heterocycles of inter­est for their biological activity, electrochemical properties, and as components of natural products (Tatu *et al.*, 2018 ▸). As part of our studies in this area, we now report the crystal structure of the title compound, C_14_H_11_N_2_O_2_SCl (Fig. 1 ▸). We believe that this is the first crystal structure to be reported of a pyrrolo­[1,2-*c*]pyrimidine and one of the few unsymmetrical ‘diaryl sulfones’ to be described.

The dihedral angle between the C1–C7/N1/N2 pyrrolo­[1,2-*c*]pyrimidine ring system (r.m.s. deviation = 0.008 Å) and the C8–C13 benzene ring is 80.2 (9)°. The rings adopt a typical diaryl sulfone ‘open-book’ conformation with respect to the sulfonyl group (Koch & Moffitt, 1951 ▸; Sime & Woodhouse, 1974 ▸; Bocelli & Rizzoli, 1990 ▸; Colquhoun, 1997 ▸; Colquhoun *et al.*, 2002 ▸; Rudolph *et al.*, 2010 ▸; Benhalima *et al.*, 2012 ▸). Notably, the torsion angles differ from the ideal 90°. Thus, the torsion angles in the title compound reveal that the *p*–*d* π overlap in the benzene ring between C8 and S1 [torsion angle C1—S1—C8—C9 = 105.19 (19)°], is favored over the p–d π overlap in the pyrrolo­[1,2-*c*]pyrimidine ring between C1 and S1 [C8—S1—C1—C2 = 110.70 (18)°], probably because the benzene ring is electron-rich relative to the pyrrolo­[1,2-*c*]pyrimidine ring. Consistent with this notion is the observation that the S1—C8 bond length [1.767 (2) Å] is slightly shorter than S1—C1 [1.773 (2) Å]. The O1—S1—O2 bond angle of 119.68 (11)° agrees with the literature values for diaryl sulfones (Sime & Woodhouse, 1974 ▸; Colquhoun, 1997 ▸; Colquhoun *et al.*, 2002 ▸; Rudolph *et al.*, 2010 ▸; Benhalima *et al.*, 2012 ▸). Likewise, the C1—S1—C8 bond angle of 105.03 (10)° is consistent with the literature data (Sime & Woodhouse, 1974 ▸; Bocelli & Rizzoli, 1990 ▸; Colquhoun *et al.*, 2002 ▸).

**Figure 2:**
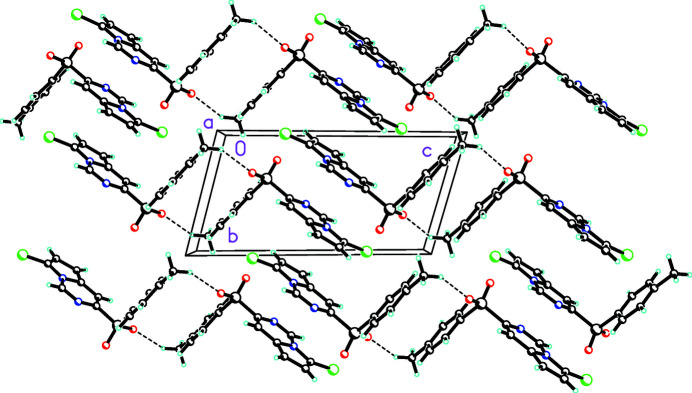
Packing diagram of the title compound viewed along the *a* axis direction showing inversion dimers linked by pairs of weak C—H⋯O inter­actions.

In the crystal, a weak C14—H14*B*⋯O2 hydrogen bond (Table 1 ▸) links two mol­ecules together in a ring face–ring face arrangement (Fig. 2 ▸). This packing motif was also observed by Sime & Woodhouse (1974 ▸) in the crystal structure of diphenyl sulfone and by Colquhoun *et al.* (2002 ▸) in the crystal structure of poly(1,4-phenyl­ene­sulfone). Several aromatic π–π stacking inter­actions between the pyrrolo­[1,2-*c*]pyrimidine rings as well as separately between the pyrrolo and pyrimidine groups (Table 2 ▸) are observed and help to consolidate the packing.

## Synthesis and crystallization

**Figure 3:**
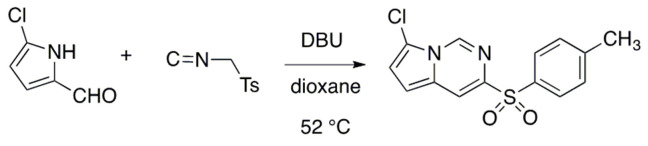
Reaction scheme.

A stirred solution of 5-chloro­pyrrole-2-carbaldehyde (Leen *et al.*, 2011 ▸) (0.023 g, 0.18 mmol) in dioxane (10 ml) was cooled in an ice bath and toluene­sulfonyl­methyl isocyanide (TosMIC) (0.046 g, 0.26 mmol) and 1,8-diazabicyclo[5.4.0]undec-7-ene (DBU) (0.04 ml) were added. The solution was then stirred for 20 h at 52°C. The reaction was quenched with 1*M* hydro­chloric acid (25 ml) and extracted with ethyl acetate (50 ml). The organic layer was washed once each with 1 *M* hydro­chloric acid (20 ml), saturated aqueous sodium bicarbonate solution (20 ml), and brine (20 ml). The organic layer was dried over anhydrous sodium sulfate, filtered over glass wool and concentrated *in vacuo*. The resulting crude product was purified by flash chromatography using 6:1 hexa­ne:ethyl acetate. Evaporation of the solvent afforded 0.036 g (66%) of 7-chloro-3-tosyl­pyrrolo­[1,2-*c*]pyrimidine as a light-yellow solid: mp 181–184°C; ^1^H NMR (500 MHz, CDCl_3_) δ 8.77 (*s*, 1H), 8.24 (*d*, *J* = 1 Hz, 1H), 7.95 (*d*, *J* = 8 Hz, 2H), 7.33 (*d*, *J* = 8 Hz, 2H), 6.92 (*d*, *J* = 4 Hz, 1H), 6.84 (*d*, *J* = 4 Hz, 1H), 2.42 (*s*, 3H); ^13^C NMR (500 MHz, CDCl_3_) δ 144.8, 141.2, 136.4, 135.9, 130.0, 129.60, 128.9, 116.9, 115.0, 110.7, 106.2, 21.8; HRMS *m/z* calculated for C_14_H_12_N_2_O_2_SCl: 307.0308, found: 307.0303. Colorless prisms suitable for X-ray crystal structure determination were recrystallized from ethanol solution. The reaction scheme is shown in Fig. 3 ▸.

## Refinement

Crystal data, data collection and structure refinement details are summarized in Table 3 ▸.

## Supplementary Material

Crystal structure: contains datablock(s) I. DOI: 10.1107/S241431462000382X/hb4342sup1.cif

Structure factors: contains datablock(s) I. DOI: 10.1107/S241431462000382X/hb4342Isup2.hkl

Supporting information file. DOI: 10.1107/S241431462000382X/hb4342Isup3.cml

CCDC reference: 1990547

Additional supporting information:  crystallographic information; 3D view; checkCIF report

## Figures and Tables

**Table 1:** Hydrogen-bond geometry (Å, °)

*D*—H⋯*A*	*D*—H	H⋯*A*	*D*⋯*A*	*D*—H⋯*A*
C14—H14*B*⋯O2^i^	0.96	2.49	3.329 (3)	146

Symmetry code: (i) 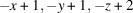 .

**Table 2:** π–π distances (Å) and angles (°) for the title compound *Cg*(*I*)—*Cg*(*J*) is the distance between ring centroids, α is dihedral angle between planes *Cg*(*I*) and *Cg*(*J*) and slippage is the distance between *Cg*(*I*) and perpendicular the projection of *Cg*(*J*) on ring *I. *Cg** (1), *Cg*(2) and *Cg*(4) are the centroids N2/C3–C6, N1/C1/C2/C3/N2/C7 and C1–C7/N1/N2 rings, respectively.

*Cg*(*I*)^*a*^	*Cg*(*J*)^*b*^	*d*[*Cg*(*I*)⋯*Cg*(*J*)]	α	slippage
*Cg*(1)	*Cg*(1)^i^	3.5758 (14)	0.00 (13)	1.169
*Cg*(1)	*Cg*(2)^i^	3.6261 (14)	0.73 (12)	1.348
*Cg*(1)	*Cg*(2)^ii^	3.6495 (14)	0.73 (12)	1.092
*Cg*(1)	*Cg*(4)^i^	3.4423 (13)	0.42 (11)	0.699
*Cg*(1)	*Cg*(4)^ii^	3.8876 (13)	0.42 (11)	1.738
*Cg*(2)	*Cg*(2)^ii^	3.9724 (13)	0.00 (10)	1.932
*Cg*(2)	*Cg*(4)^ii^	3.6901 (12)	0.32 (9)	1.233
*Cg*(4)	*Cg*(4)^i^	3.7241 (11)	0.00 (7)	1.586
*Cg*(4)	*Cg*(4)^ii^	3.6280 (11)	0.00 (7)	1.010

Symmetry codes: (i) 1 − *x*, −*y*, 1 − *z*; (ii) −*x*, 1 − *y*, 1 − *z*.

**Table 3:** Experimental details

Crystal data	
Chemical formula	C_14_H_11_ClN_2_O_2_S
*M*_r_	306.76
Crystal system, space group	Triclinic, *P* 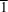
Temperature (K)	173
*a*, *b*, *c* (Å)	7.2285 (5), 7.2385 (5), 13.7586 (11)
α, β, γ (°)	102.192 (7), 99.616 (6), 104.149 (6)
*V* (Å^3^)	663.91 (9)
*Z*	2
Radiation type	Cu *K*α
μ (mm^−1^)	4.05
Crystal size (mm)	0.16 × 0.13 × 0.08

Data collection	
Diffractometer	Rigaku Oxford Diffraction Eos, Gemini
Absorption correction	Multi-scan
*T*_min_, *T*_max_	0.748, 1.000
No. of measured, independent and observed [*I* > 2σ(*I*)] reflections	3839, 2498, 2177
*R*_int_	0.036
(sin θ/λ)_max_ (Å^−1^)	0.614

Refinement	
*R*[*F* ^2^ > 2σ(*F* ^2^)], *wR*(*F* ^2^), *S*	0.043, 0.114, 1.04
No. of reflections	2498
No. of parameters	183
H-atom treatment	H-atom parameters constrained
Δρ_max_, Δρ_min_ (e Å^−3^)	0.53, −0.31

Computer programs: *CrysAlis PRO* (Rigaku OD, 2019 ▸), SHELXT (Sheldrick, 2015*a* ▸), *SHELXL* (Sheldrick, 2015*b* ▸) and *OLEX2* (Dolomanov *et al.*, 2009 ▸).

## full crystallographic data

### Crystal data

**Table:**

C_14_H_11_ClN_2_O_2_S	*Z* = 2
*M**_r_* = 306.76	*F*(000) = 316
Triclinic, *P*1	*D*_x_ = 1.535 Mg m^−^^3^
*a* = 7.2285 (5) Å	Cu *K*α radiation, λ = 1.54184 Å
*b* = 7.2385 (5) Å	Cell parameters from 1885 reflections
*c* = 13.7586 (11) Å	θ = 6.5–71.4°
α = 102.192 (7)°	µ = 4.05 mm^−^^1^
β = 99.616 (6)°	*T* = 173 K
γ = 104.149 (6)°	Prism, colourless
*V* = 663.91 (9) Å^3^	0.16 × 0.13 × 0.08 mm

### Data collection

**Table:**

Rigaku-Oxford Diffraction Eos, Gemini diffractometer	2498 independent reflections
Radiation source: fine-focus sealed X-ray tube	2177 reflections with *I* > 2σ(*I*)
Detector resolution: 16.0416 pixels mm^-1^	*R*_int_ = 0.036
ω scans	θ_max_ = 71.2°, θ_min_ = 3.4°
Absorption correction: multi-scan	*h* = −5→8
*T*_min_ = 0.748, *T*_max_ = 1.000	*k* = −8→8
3839 measured reflections	*l* = −16→16

### Refinement

**Table:**

Refinement on *F*^2^	Hydrogen site location: inferred from neighbouring sites
Least-squares matrix: full	H-atom parameters constrained
*R*[*F*^2^ > 2σ(*F*^2^)] = 0.043	*w* = 1/[σ^2^(*F*_o_^2^) + (0.0554*P*)^2^] where *P* = (*F*_o_^2^ + 2*F*_c_^2^)/3
*wR*(*F*^2^) = 0.114	(Δ/σ)_max_ < 0.001
*S* = 1.04	Δρ_max_ = 0.53 e Å^−^^3^
2498 reflections	Δρ_min_ = −0.31 e Å^−^^3^
183 parameters	Extinction correction: SHELXL-2018/1 (Sheldrick 2015), Fc^*^=kFc[1+0.001xFc^2^λ^3^/sin(2θ)]^-1/4^
0 restraints	Extinction coefficient: 0.0018 (6)
Primary atom site location: dual	

### Special details

**Table:**

Geometry. All esds (except the esd in the dihedral angle between two l.s. planes) are estimated using the full covariance matrix. The cell esds are taken into account individually in the estimation of esds in distances, angles and torsion angles; correlations between esds in cell parameters are only used when they are defined by crystal symmetry. An approximate (isotropic) treatment of cell esds is used for estimating esds involving l.s. planes.
Refinement. All of the H atoms were placed in their calculated positions and then refined with atom-H lengths of 0.93 Å (CH); 0.96 Å (CH_3_) using the riding model with *U*_iso_ (H) = 1.2 (CH) or 1.5 times *U*_eq_ (CH_3_) of the parent atom. The idealized methyl group was refined as a rotating group.

### Fractional atomic coordinates and isotropic or equivalent isotropic displacement parameters (Å^2^)

**Table:**

	*x*	*y*	*z*	*U*_iso_*/*U*_eq_	
Cl1	0.01870 (9)	0.02376 (9)	0.27155 (4)	0.03578 (19)	
S1	0.40553 (8)	0.62315 (7)	0.76558 (4)	0.02404 (18)	
O1	0.5458 (3)	0.7802 (2)	0.74516 (13)	0.0334 (4)	
O2	0.2700 (3)	0.6692 (3)	0.82618 (13)	0.0340 (4)	
N1	0.3648 (3)	0.4137 (3)	0.57537 (14)	0.0250 (4)	
N2	0.0639 (3)	0.2272 (3)	0.46404 (13)	0.0216 (4)	
C1	0.2605 (3)	0.4612 (3)	0.64732 (16)	0.0221 (4)	
C2	0.0627 (3)	0.3971 (3)	0.63311 (16)	0.0231 (4)	
H2	0.001144	0.434521	0.685034	0.028*	
C3	−0.0462 (3)	0.2721 (3)	0.53723 (17)	0.0228 (4)	
C4	−0.2410 (3)	0.1749 (3)	0.49171 (18)	0.0282 (5)	
H4	−0.346453	0.178047	0.521972	0.034*	
C5	−0.2517 (3)	0.0705 (3)	0.39148 (18)	0.0289 (5)	
H5	−0.365477	−0.008063	0.343710	0.035*	
C6	−0.0663 (3)	0.1047 (3)	0.37665 (16)	0.0257 (5)	
C7	0.2645 (3)	0.3009 (3)	0.48672 (16)	0.0235 (4)	
H7	0.331004	0.268421	0.436325	0.028*	
C8	0.5354 (3)	0.4809 (3)	0.82241 (16)	0.0253 (4)	
C9	0.7327 (4)	0.5134 (3)	0.82356 (17)	0.0293 (5)	
H9	0.796540	0.608266	0.794615	0.035*	
C10	0.8339 (4)	0.4003 (4)	0.86927 (19)	0.0316 (5)	
H10	0.966752	0.421378	0.871207	0.038*	
C11	0.7390 (4)	0.2571 (3)	0.91184 (16)	0.0269 (5)	
C12	0.5414 (4)	0.2299 (4)	0.90997 (19)	0.0331 (5)	
H12	0.476588	0.134692	0.938446	0.040*	
C13	0.4392 (4)	0.3421 (4)	0.86641 (19)	0.0326 (5)	
H13	0.307487	0.324508	0.866706	0.039*	
C14	0.8477 (4)	0.1336 (4)	0.95979 (18)	0.0335 (5)	
H14A	0.794694	−0.002190	0.921296	0.050*	
H14B	0.833361	0.145628	1.028789	0.050*	
H14C	0.984104	0.178670	0.959859	0.050*	

### Atomic displacement parameters (Å^2^)

**Table:**

	*U*^11^	*U*^22^	*U*^33^	*U*^12^	*U*^13^	*U*^23^
Cl1	0.0431 (4)	0.0340 (3)	0.0238 (3)	0.0029 (2)	0.0081 (2)	0.0033 (2)
S1	0.0260 (3)	0.0219 (3)	0.0216 (3)	0.0068 (2)	0.0011 (2)	0.00387 (19)
O1	0.0329 (9)	0.0238 (8)	0.0372 (9)	0.0025 (7)	−0.0034 (7)	0.0107 (7)
O2	0.0357 (9)	0.0376 (9)	0.0269 (8)	0.0160 (7)	0.0033 (7)	0.0016 (7)
N1	0.0239 (9)	0.0272 (9)	0.0235 (9)	0.0050 (7)	0.0050 (7)	0.0091 (7)
N2	0.0240 (9)	0.0212 (8)	0.0200 (8)	0.0054 (7)	0.0031 (7)	0.0089 (7)
C1	0.0240 (10)	0.0209 (10)	0.0214 (10)	0.0054 (8)	0.0040 (8)	0.0080 (8)
C2	0.0271 (11)	0.0234 (10)	0.0228 (10)	0.0108 (8)	0.0080 (8)	0.0090 (8)
C3	0.0220 (10)	0.0247 (10)	0.0263 (10)	0.0093 (8)	0.0067 (8)	0.0124 (8)
C4	0.0207 (10)	0.0310 (12)	0.0338 (12)	0.0076 (9)	0.0030 (9)	0.0128 (9)
C5	0.0276 (11)	0.0257 (11)	0.0302 (11)	0.0044 (9)	−0.0018 (9)	0.0109 (9)
C6	0.0309 (11)	0.0210 (10)	0.0230 (10)	0.0050 (8)	0.0021 (9)	0.0070 (8)
C7	0.0230 (10)	0.0236 (10)	0.0244 (10)	0.0056 (8)	0.0069 (8)	0.0077 (8)
C8	0.0275 (11)	0.0241 (10)	0.0216 (10)	0.0063 (8)	−0.0004 (8)	0.0065 (8)
C9	0.0342 (12)	0.0299 (11)	0.0278 (11)	0.0096 (9)	0.0111 (9)	0.0124 (9)
C10	0.0262 (11)	0.0391 (13)	0.0331 (11)	0.0120 (10)	0.0091 (9)	0.0124 (10)
C11	0.0331 (12)	0.0260 (11)	0.0193 (9)	0.0095 (9)	0.0011 (8)	0.0038 (8)
C12	0.0322 (12)	0.0326 (12)	0.0360 (12)	0.0064 (10)	0.0074 (10)	0.0161 (10)
C13	0.0248 (11)	0.0350 (12)	0.0375 (12)	0.0047 (9)	0.0054 (9)	0.0145 (10)
C14	0.0435 (14)	0.0328 (12)	0.0275 (11)	0.0179 (11)	0.0058 (10)	0.0088 (9)

### Geometric parameters (Å, º)

**Table:**

Cl1—C6	1.711 (2)	C2—C3	1.407 (3)
S1—O1	1.4370 (18)	C3—C4	1.380 (3)
S1—O2	1.4429 (18)	C4—C5	1.405 (3)
S1—C1	1.773 (2)	C5—C6	1.360 (3)
S1—C8	1.767 (2)	C8—C9	1.384 (3)
N1—C1	1.378 (3)	C8—C13	1.384 (3)
N1—C7	1.294 (3)	C9—C10	1.399 (3)
N2—C3	1.417 (3)	C10—C11	1.390 (3)
N2—C6	1.371 (3)	C11—C12	1.389 (4)
N2—C7	1.373 (3)	C11—C14	1.507 (3)
C1—C2	1.358 (3)	C12—C13	1.384 (4)
			
O1—S1—O2	119.68 (11)	C3—C4—C5	107.8 (2)
O1—S1—C1	108.51 (10)	C6—C5—C4	108.0 (2)
O1—S1—C8	107.90 (11)	N2—C6—Cl1	119.51 (18)
O2—S1—C1	106.24 (11)	C5—C6—Cl1	130.89 (18)
O2—S1—C8	108.56 (11)	C5—C6—N2	109.6 (2)
C8—S1—C1	105.03 (10)	N1—C7—N2	122.67 (19)
C7—N1—C1	116.87 (19)	C9—C8—S1	119.13 (17)
C6—N2—C3	107.32 (18)	C13—C8—S1	119.48 (18)
C6—N2—C7	131.24 (19)	C13—C8—C9	121.4 (2)
C7—N2—C3	121.44 (18)	C8—C9—C10	118.5 (2)
N1—C1—S1	114.68 (16)	C11—C10—C9	121.1 (2)
C2—C1—S1	119.95 (16)	C10—C11—C14	121.0 (2)
C2—C1—N1	125.37 (19)	C12—C11—C10	118.8 (2)
C1—C2—C3	117.78 (19)	C12—C11—C14	120.2 (2)
C2—C3—N2	115.87 (19)	C13—C12—C11	121.1 (2)
C4—C3—N2	107.3 (2)	C8—C13—C12	119.2 (2)
C4—C3—C2	136.8 (2)		
			
S1—C1—C2—C3	178.70 (15)	C3—C4—C5—C6	−0.2 (3)
S1—C8—C9—C10	179.69 (17)	C4—C5—C6—Cl1	−179.09 (17)
S1—C8—C13—C12	179.40 (19)	C4—C5—C6—N2	0.2 (2)
O1—S1—C1—N1	44.81 (18)	C6—N2—C3—C2	179.87 (17)
O1—S1—C1—C2	−134.12 (16)	C6—N2—C3—C4	0.0 (2)
O1—S1—C8—C9	−10.4 (2)	C6—N2—C7—N1	−178.8 (2)
O1—S1—C8—C13	168.42 (19)	C7—N1—C1—S1	−177.86 (15)
O2—S1—C1—N1	174.70 (15)	C7—N1—C1—C2	1.0 (3)
O2—S1—C1—C2	−4.23 (19)	C7—N2—C3—C2	0.5 (3)
O2—S1—C8—C9	−141.51 (19)	C7—N2—C3—C4	−179.37 (18)
O2—S1—C8—C13	37.3 (2)	C7—N2—C6—Cl1	−1.5 (3)
N1—C1—C2—C3	−0.1 (3)	C7—N2—C6—C5	179.1 (2)
N2—C3—C4—C5	0.2 (2)	C8—S1—C1—N1	−70.37 (17)
C1—S1—C8—C9	105.19 (19)	C8—S1—C1—C2	110.70 (18)
C1—S1—C8—C13	−76.0 (2)	C8—C9—C10—C11	0.6 (4)
C1—N1—C7—N2	−1.1 (3)	C9—C8—C13—C12	−1.8 (4)
C1—C2—C3—N2	−0.6 (3)	C9—C10—C11—C12	−1.1 (4)
C1—C2—C3—C4	179.2 (2)	C9—C10—C11—C14	179.2 (2)
C2—C3—C4—C5	−179.7 (2)	C10—C11—C12—C13	0.2 (4)
C3—N2—C6—Cl1	179.28 (14)	C11—C12—C13—C8	1.2 (4)
C3—N2—C6—C5	−0.1 (2)	C13—C8—C9—C10	0.9 (3)
C3—N2—C7—N1	0.4 (3)	C14—C11—C12—C13	179.9 (2)

### Hydrogen-bond geometry (Å, º)

**Table:**

*D*—H···*A*	*D*—H	H···*A*	*D*···*A*	*D*—H···*A*
C14—H14*B*···O2^i^	0.96	2.49	3.329 (3)	146

Symmetry code: (i) −*x*+1, −*y*+1, −*z*+2.
